# Supplementary material for: COVID-19 vaccination in Nigeria: A rapid review of vaccine acceptance rate and the associated factors
Source: PLoS One. 2022 May 11;17(5):e0267691. doi: 10.1371/journal.pone.0267691 (PMC9094528; doi:10.1371/journal.pone.0267691)
Supplement: Attachment — Submitted filename: PONE-D-21-39940_AA.pdf [file pone.0267691.s002.pdf]

PLOS ONE

COVID-19 vaccination in Nigeria: a rapid review of vaccine acceptance rate and associated factors

--Manuscript Draft--

**Manuscript Number:**	PONE-D-21-39940
**Article Type:**	Research Article
**Full Title:**	COVID-19 vaccination in Nigeria: a rapid review of vaccine acceptance rate and associated factors
**Short Title:**	COVID-19 vaccine acceptance rate in Nigeria
**Corresponding Author:**	Olumide Abiodun<br>Babcock University Benjamin S Carson Senior School of Medicine<br>NIGERIA
**Keywords:**	vaccine, vaccination acceptance rates, COVID-19, Review, Factors, Unwillingness.
**Abstract:**	Vaccine acceptance among a large population of people can determine the successful control of the COVID-19 pandemic. We aimed to assess the COVID 19 vaccine acceptance rate and to identify the predicting factors to the non-acceptance of the vaccine in Nigeria up to date. In line with this, PubMed, Web of Science, Cochrane Library, and Embase databases until October 2021 were searched. Ten articles with 9,287 individuals met the inclusion criteria and formed the basis for the final COVID-19 acceptance estimates.<br>A total of ten peer-reviewed articles were reviewed. The vaccine acceptance rate ranged from 20.0% to 58.2%. among adults across the six geopolitical zones of the country. Non-acceptance of the vaccine was found to be a result of ~~disbelief~~, propaganda, adverse effect concerns, and conspiracy the[illegible]s.<br>National, community and individual-level interventions need to be developed to improve the COVID-19 acceptance rate ~~of~~ the country. Greater efforts could be put in place to address the issues of concern leading to the unwillingness of the people to receive the COVID-19 vaccine.
**Order of Authors:**	Oluwatosin Olu-Abiodun, BNSc. MNSc.
	Olumide Abiodun, MBChB, FWACP
**Additional Information:**	
**Question**	**Response**
**Financial Disclosure**<br><br>Enter a financial disclosure statement that describes the sources of funding for the work included in this submission. Review the submission guidelines for detailed requirements. View published research articles from *PLOS ONE* for specific examples.<br><br>This statement is required for submission and **will appear in the published article** if the submission is accepted. Please make sure it is accurate.	Nil

**Unfunded studies**<br>Enter: *The author(s) received no specific funding for this work.*<br><br>**Funded studies**<br>Enter a statement with the following details:<br>• Initials of the authors who received each award<br>• Grant numbers awarded to each author<br>• The full name of each funder<br>• URL of each funder website<br>• Did the sponsors or funders play any role in the study design, data collection and analysis, decision to publish, or preparation of the manuscript?<br>• **NO** - Include this sentence at the end of your statement: *The funders had no role in study design, data collection and analysis, decision to publish, or preparation of the manuscript.*<br>• **YES** - Specify the role(s) played.<br><br>* typeset	
**Competing Interests**<br><br>Use the instructions below to enter a competing interest statement for this submission. On behalf of all authors, disclose any competing interests that could be perceived to bias this work—acknowledging all financial support and any other relevant financial or non-financial competing interests.<br><br>This statement is required for submission and **will appear in the published article** if the submission is accepted. Please make sure it is accurate and that any funding sources listed in your Funding Information later in the submission form are also declared in your Financial Disclosure statement.<br><br>View published research articles from *PLOS ONE* for specific examples.	The authors declare no conflict of interest

**NO authors have competing interests**<br><br>Enter: *The authors have declared that no competing interests exist.*<br><br>**Authors with competing interests**<br><br>Enter competing interest details beginning with this statement:<br><br>*I have read the journal's policy and the authors of this manuscript have the following competing interests: [insert competing interests here]*<br><br>* typeset	
**Ethics Statement**<br><br>Enter an ethics statement for this submission. This statement is required if the study involved:<br><br>• Human participants<br>• Human specimens or tissue<br>• Vertebrate animals or cephalopods<br>• Vertebrate embryos or tissues<br>• Field research<br><br>Write "N/A" if the submission does not require an ethics statement.<br><br>General guidance is provided below. Consult the submission guidelines for detailed instructions. **Make sure that all information entered here is included in the Methods section of the manuscript.**	Ethics statement<br>This article reports the review of published publicly available manuscripts and did not require the collection of original data or interaction with human participants in any form, therefore, IRB was not obtained.

Powered by Editorial Manager® and ProduXion Manager® from Aries Systems Corporation

**Format for specific study types**<br><br>**Human Subject Research (involving human participants and/or tissue)**<br>• Give the name of the institutional review board or ethics committee that approved the study<br>• Include the approval number and/or a statement indicating approval of this research<br>• Indicate the form of consent obtained (written/oral) or the reason that consent was not obtained (e.g. the data were analyzed anonymously)<br><br>**Animal Research (involving vertebrate animals, embryos or tissues)**<br>• Provide the name of the Institutional Animal Care and Use Committee (IACUC) or other relevant ethics board that reviewed the study protocol, and indicate whether they approved this research or granted a formal waiver of ethical approval<br>• Include an approval number if one was obtained<br>• If the study involved *non-human primates*, add *additional details* about animal welfare and steps taken to ameliorate suffering<br>• If anesthesia, euthanasia, or any kind of animal sacrifice is part of the study, include briefly which substances and/or methods were applied<br><br>**Field Research**<br><br>Include the following details if this study involves the collection of plant, animal, or other materials from a natural setting:<br>• Field permit number<br>• Name of the institution or relevant body that granted permission	
**Data Availability**<br><br>Authors are required to make all data underlying the findings described fully available, without restriction, and from the time of publication. PLOS allows rare exceptions to address legal and ethical concerns. See the PLOS Data Policy and FAQ for detailed information.	Yes - all data are fully available without restriction

A Data Availability Statement describing where the data can be found is required at submission. Your answers to this question constitute the Data Availability Statement and **will be published in the article**, if accepted.<br><br>**Important:** Stating 'data available on request from the author' is not sufficient. If your data are only available upon request, select 'No' for the first question and explain your exceptional situation in the text box.<br><br>Do the authors confirm that all data underlying the findings described in their manuscript are fully available without restriction?	
**Describe where the data may be found in full sentences. If you are copying our sample text, replace any instances of XXX with the appropriate details.**<br><br>• If the data are **held or will be held in a public repository**, include URLs, accession numbers or DOIs. If this information will only be available after acceptance, indicate this by ticking the box below. For example: *All XXX files are available from the XXX database (accession number(s) XXX, XXX.).*<br>• If the data are all contained **within the manuscript and/or Supporting Information files**, enter the following: *All relevant data are within the manuscript and its Supporting Information files.*<br>• If neither of these applies but you are able to provide **details of access elsewhere**, with or without limitations, please do so. For example:<br><br>*Data cannot be shared publicly because of [XXX]. Data are available from the XXX Institutional Data Access / Ethics Committee (contact via XXX) for researchers who meet the criteria for access to confidential data.*<br><br>*The data underlying the results presented in the study are available from (include the name of the third party*	All data used in this review are publicly available

and contact information or URL).<br>• This text is appropriate if the data are owned by a third party and authors do not have permission to share the data.<br><br>* typeset	
Additional data availability information:	Tick here if the URLs/accession numbers/DOIs will be available only after acceptance of the manuscript for publication so that we can ensure their inclusion before publication.

Powered by Editorial Manager® and ProduXion Manager® from Aries Systems Corporation

Manuscript
Click here to access/download;Manuscript;Manuscript PONE.docx

COVID-19 vaccination in Nigeria: a rapid review of vaccine acceptance rate and associated factors

Oluwatosin Olu-Abiodun[1], Olumide Abiodun[2*]

1. Department of Community Health Nursing, Babcock University, Ilishan, Nigeria. tmtosin@yahoo.co.uk
2. Department of Community Medicine, Babcock University, Ilishan, Nigeria. abioduno@babcock.edu.ng

*Correspondence: Olumide Abiodun
Department of Community Medicine, Babcock University, Ilishan, Nigeria
abioduno@babcock.edu.ng +2347038569725

Abstract

Vaccine acceptance among a large population of people can determine the successful control of the COVID-19 pandemic. We aimed to assess the COVID-19 vaccine acceptance rate and to identify the predicting factors to the non-acceptance of the vaccine in Nigeria up to date. In line with this, PubMed, Web of Science, Cochrane Library, and Embase databases until October 2021 were searched in this rapid review. Ten articles with 9,287 individuals met the inclusion criteria and formed the basis for the final COVID-19 acceptance estimates.

A total of ten peer-reviewed articles were reviewed. The vaccine acceptance rate ranged from 20.0% to 58.2%. among adults across the six geopolitical zones of the country. Non-acceptance of the vaccine was found to be a result of disbelief, propaganda, adverse effect concerns, and conspiracy theories.

National, community and individual-level interventions need to be developed to improve the COVID-19 acceptance rate of the country. Greater efforts could be put in place to address the issues of concern leading to the unwillingness of the people to receive the COVID-19 vaccine.

Keywords: vaccine, vaccination acceptance rates, COVID-19, Review, Factors, Unwillingness.

Introduction

Severe acute respiratory syndrome (SARS) first appeared in November 2002 in the Guangdong province of southern China (WHO, 2015). It subsequently spread, and an epidemic of SARS affected 26 countries and resulted in more than 8000 cases in 2003 (WHO, 2015). However, from 2004, no known cases of SARS were reported anywhere in the world (CDC, 2014), until December 2019, when a new strain of severe acute respiratory syndrome coronavirus 2 (SARS-CoV-2) was first reported in Wuhan province of China, the epicenter, and have since been declared by WHO as a global pandemic spreading at a very fast rate across the globe. It was first reported in Lagos, Nigeria on February 27, 2020 (NCDC, 2020).

As of November 2021, over two hundred and sixty-two million confirmed cases of COVID-19 had been reported globally, with over five million associated deaths and this has led to huge psychological, sociological, and economic turmoil around the globe (WHO, 2021). In Nigeria, over two hundred and fourteen thousand cases have been reported with over two thousand deaths (NCDC, 2021). Unfortunately, many Nigerians do not believe in the existence of the virus. At the peak of the pandemic in 2020, far-reaching measures such as extensive testing, nationwide lockdown, social distancing, use of face masks, and isolation of infected persons were put in place to curb the further spread of COVID-19. However, these measures were not enough to contain the spread of the virus and it was not easy to enforce among the general populace.

The coronavirus disease pandemic of 2019 (COVID-19) is probably humanity's greatest challenge. Normalcy before the pandemic is unlikely to return until a safe and effective vaccination is successfully implemented. Vaccines strengthen the immune system by utilizing the body's defenses to build resistance to specific pathogens. Vaccines teach the immune system to make antibodies in the same way that it does when the body is exposed to a disease. Not too long after the emergence of the pandemic, lots of effective and safe vaccines against COVID-19 were rolled out globally. There are currently at least thirteen COVID-19 vaccines approved for early or limited use, and twenty-seven others are in large scale, Phase III, final trials (as of 18 March 2021) (Samaranayake & Fakhruddin, 2021; WHO, 2021).

Since there is no specific treatment for COVID-19, vaccination is still one of the most effective means of preventing the disease. Nevertheless, the progress made by the development of safe and effective vaccines, there are still cogent issues that need to be addressed regarding COVID-19 vaccines which have been accompanied by widespread misinformation, a plethora of conspiracy theories surrounding the virus, and vaccine development by political and religious leaders that has resulted into non-acceptance of the vaccine by many people. Across the world, it has been reported that people who were fully vaccinated died of COVID-19 associated symptoms, which has also deepened the public uncertainty about the safety and effectiveness of the vaccines.

Ending the SARS-CoV-2 (COVID-19) pandemic will require unprecedented collective action, on a global, national, and sub-national scale. In addition to social distancing and practicing other pro-social health behaviors (e.g., wearing masks in public places), people must choose to receive a COVID-19 vaccine. To achieve herd immunity against COVID-19, Nigeria had set an ambitious goal of vaccinating 40 percent of its over 200 million populations before the end of 2021, and 70 percent by the end of 2022 (NCDC, 2021). About 70 percent of the total population needs to receive the COVID-19 vaccines to completely eradicate

the virus, and to vaccinate 40 percent of Nigeria's 200 million citizens means 80 million people would have received their full doses by the end of the year.

COVID-19 vaccination rate

As of 28 November 2021, a total of 7.81B vaccine doses have been administered globally (WHO, 2021). In Nigeria, as of 19 November 2021, 6,021,560 people have received their first dose of the COVID-19 vaccine, and only 3,369,628 people have received their second dose, bringing the unvaccinated population to approximately 202,009,520 people (97.15%) (NPHCDA, 2021).

Despite the desperate need for interventions from vaccines, there is still considerable apathy and hesitancy around the acceptance and use of the available, approved vaccines in many places around the world, in Africa and Nigeria, especially. Going by the current vaccination pace, however, less than nine million people should have been vaccinated in Nigeria by the end of 2021. The figure is less than 15 percent of the 80 million citizens that were targeted. This is more worrisome because even health workers and the at most risk population (elderly and people with co-morbidities) appear to be reluctant to take a jab. At this rate, it's most unlikely that the target will be met and the spread might also be on the increase thereby declining the population growth of the country.

TABLE 1: Common COIVD-19 Vaccines (Samaranayake, Chang, & Panduwawala, 2021)

Name	Platform	Required doses	Interval btw doses	Efficacy against original strain (%)	Efficacy against variants	Preventing hospitalization (%)	Protection from severe disease (%)	Protection from milder disease (%)
PFIZER	mRNA in lipid nanoparticles	2	21days	95	UK, South African, Latin American	100	100	94.1
Moderna	mRNA in lipid nanoparticles	2	28days	95	UK, South African, Latin American	100	100	95.0
AstraZeneca/ Oxford	Adenovirus based	2	Btw 4 and 12weeks	70	UK; South African, Latin American (low)	100	100	90.
Johnson & Johnson	Non replicating human adenovirus based/DN	1	NA	66-77	UK; South African, Latin American (low)	85	85	USA:72.0 Latin America: 66 South Africa:57
Sputnik V	Non replicating chimp adenovirus based/DNA	2	21Days	90	No data	100	100	USSR: 91.4
Sinovac	Inactivated SARS-CoV-2	2	28days	50-90	50% against Latin American Strain	100	-	-
Novavax	Protein based/subunit (RBDMatrix M adjuvant)	2	16 days	89	UK, South Africa	100	-	UK: 89.3 South Africa: 60

COVID-19 vaccine acceptance rate

COVID-19 acceptance rates worldwide and in Africa have been surveyed and reported in previous studies. The proportion of willingness to accept COVID-19 vaccination drawn from pooled prevalence acceptance rates across the world has been rated below 60% (Sallam, 2021) and 48.93% across Africa (Wake, 2021). However, some of the problems were not explored. By identifying the factors responsible for unwillingness to accept the vaccine, we can help to inform the development of evidence-based guidelines to effectively improve the COVID-19 uptake. Hence, the current review sought to estimate the COVID-19 acceptance rate and identify the factors responsible for unwillingness to accept the vaccine in Nigeria.

Materials and methods

This is a rapid review of studies conducted in Nigeria. Published papers in African Journals Online, PubMed, Cochrane Review, HINARI, EMBASE, and Google Scholar were used to retrieve related articles. During this review, the search was done by using keywords such as; "unwillingness," "acceptance,"," COVID-19 vaccination," "COVID-19," "SARS-CoV-2," "vaccine," and "Nigeria." To integrate these keywords; Boolean operators "AND" and "OR" were used. Only studies published in the English language up to November 2021, that met the inclusion criteria were considered in this review.

The inclusion criteria were: (1) peer-reviewed published articles indexed in PubMed; (2) cross-sectional studies among the general population, healthcare workers, students, or parents/guardians); (3) the major aim of the study was to evaluate COVID-19 vaccine acceptance/hesitancy; and (4) publication language was English. The exclusion criteria were: (1) unpublished manuscripts (preprints); (2) the articles that did not aim to evaluate COVID-19 vaccine acceptance/hesitancy, and (3) publication language was not English.

Ethics statement

This article reports the review of published publicly available manuscripts and did not require the collection of original data or interaction with human participants in any form, therefore, IRB was not obtained. Since the study did not involve participants, consent was neither required nor feasible.

Data extraction

Screening of titles and abstracts was conducted, followed by data extraction for the following items: author, date of survey, states in which the survey was conducted, the target population for the survey (e.g., the general public, healthcare workers, and students), the total number of respondents, COVID-19 vaccine acceptance rate and factors that predict non-acceptance of the vaccine.

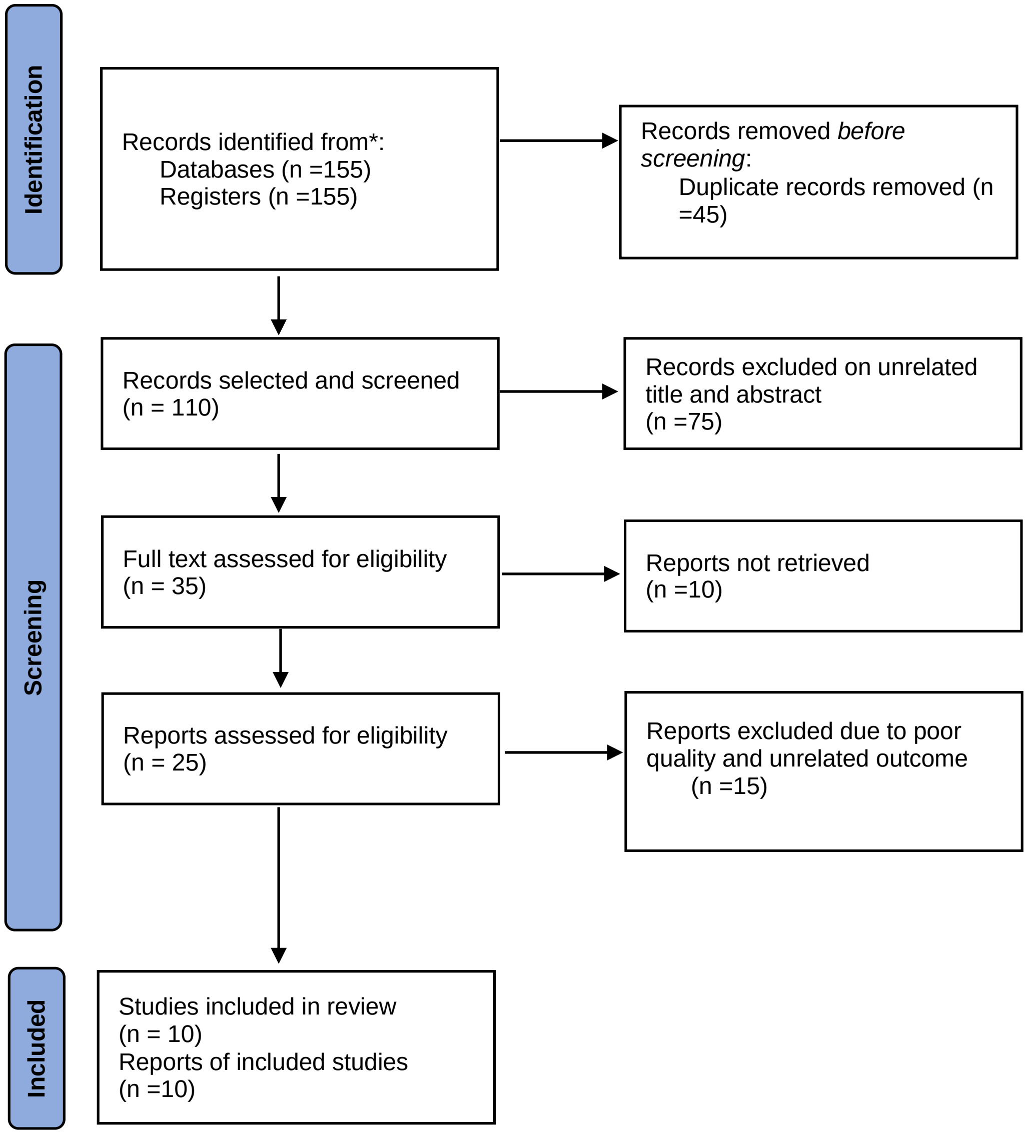

Figure 1: Study flow chart

Results

Published papers in African Journals Online, PubMed, Cochrane Review, HINARI, EMBASE, and Google Scholar were used to retrieve related articles. A total of 155 articles were found. 45 articles were excluded because of duplication, 75 were excluded by unrelated titles and abstract, ten were excluded due to inability to access the full text. 25 articles were checked for eligibility and 15 were excluded due to poor quality and an unrelated outcome variable. A total of ten articles met the eligibility criteria and were included in this review.

Characteristics of the papers included in this review

A total of ten published papers were analyzed in this review. These studies comprised surveys on COVID -19 Vaccine acceptance from across Nigeria with over 11,500 participants, up till March 2021. The survey was mostly done in the southeast of the country. The studies were cross-sectional studies with mainly online recruitment of respondents. The largest sample size was 1740 from a study carried out in Ondo, Edo, and Delta state, while the smallest sample size was 339 from a study carried out in Enugu, Ebonyi, and Anambra State in the South East region. Most of the recruited participants in the studies are adults across the nation. Dates of survey distribution ranged from May 2020 to March 2021.

Table 2; Characteristics of the studies included in the rapid review on the level of acceptance towards COVID-19 among Nigerians

s/n	Study	Stata	Date of survey	Number of participants	Target population	Acceptance rate	Factors
1	(Adejumo et al., 2021)	Ondo<br>Edo<br>Delta	Oct 2020	1740	Health workers	55.5%	Vaccines might not be safe,
2	(Uzochukwu et al., 2021)	Anambra	Jan to Feb 2021	349	University students and staffs	34.7%	Disbelief, poor knowledge of the technology platforms used in the design and development of the vaccine, Deficient data about vaccine adverse effect, Religious inclination
3	(Enitan et al., 2020)	Across six geopolitical Zones	May 2020	465	Adults	20.0%	Disbeliefs, conspiracy theories, and fear of the unknown
4	(Adigwe, 2021)	Abuja	Jan 2021	1767	Adults	47.1%	Side effects, vaccine safety, and risk concern
5	(Olomofe et al., 2021)	Across five geopolitical Zones	June to July 2020	776	Adults	58.2%	Fear of the unknown, conspiracy theories
6	(Tobin, Okonofua, & Azeke, 2021)	Across 36 States	July to August 2020	1228	Nigerian adults	50.2%	Misinformation, conspiracy theories, lack of trust in the government, Religious inclination
7	(Amuzie et al., 2021)	Abia	Mar 2021	422	Health workers	45.6%	Lack of trust, misinformation, conspiracy theories
8	(Allagoa et al., 2021)	Bayelsa	Jan to Feb 2021	1000	Patients	24.6%	Disbelief, conspiracy theories, safety issues, and religious sentiments
9	(Robinson, Wilson, Eleki, & Wonodi, 2021)	Across six geopolitical zones	Dec 2020 to Jan 2021	1094	Health workers	32.5%	Effectiveness, fear of the known, and safety concerns.
10	(Iliyasu et al., 2021)	Kano	Mar 2021	446	Adults	51.1%	Vaccine safety and rumors

COVID-19 vaccine acceptance rate

The results of the COVID 19 vaccine acceptance rates in different studies included in this review are shown in table 2. The vaccine acceptance rate ranged from 20.0% to 58.2%. Classified per the study, the highest vaccine acceptance rate was 58.2% in a study across the six geopolitical zones of Nigeria (Olomofe et al., 2021), 55.5% in Ondo, Edo, and Delta (Adejumo et al., 2021), 51.1% in Kano (Iliyasu et al., 2021), and 50.2% across the six geopolitical zones of Nigeria (Tobin et al., 2021). On the contrary, the lowest acceptance rate was 20.0% across the six geopolitical zones (Enitan et al., 2020), 24.6% from Bayelsa state (Allagoa et al., 2021), and 32.52% across the six geopolitical zones of Nigeria (Robinson et al., 2021). This followed by 34.7% in Anambra (Uzochukwu et al., 2021), 45.6% in Abia (Amuzie et al., 2021), and 47.1% in Abuja (Adigwe, 2021).

Among health workers, the vaccine acceptance rate was between 32.5 and 55.5%. For the three studies conducted on health workers, two surveys reported vaccine acceptance rate below 50% with the highest of 55.5% in Ondo, Edo, and Delta (Adejumo et al., 2021) ~~with~~ the lowest vaccine acceptance rate (32.5%) reported among health workers across the six geopolitical zones of the country (Robinson et al., 2021).

Among the adult population, the acceptance rate was between 20.0% and 58.2%. For the five studies conducted among adults, the vaccine acceptance rate was a little above 50% except for the study that had a 20.0% acceptance rate (Enitan et al., 2020). Among university staff and students, a prevalence rate of 34.7% was reported (Uzochukwu et al., 2021), also, a 24.6% acceptance rate was reported among patients that presented for management at a tertiary health care facility (Allagoa et al., 2021).

Variations in COVID-19 acceptance rate across the country in populations with multiple survey studies.

Among populations with multiple studies, the following changes in COVID-19 acceptance rates were observed. Among healthcare workers, the acceptance rate was 55.5% in October 2020, 32.5% in January 2021, and 45.6% in March 2021. Among adults, the acceptance rate was 20.0% in May 2020, 58.2% in July 2020, 50.2% in August 2020, 51.1% in February 2021, and 45.6% in March 2021.

Factors responsible for non-acceptance of COVID-19 vaccines.

The studies explored the factors for the refusal of the respondents to accept COVID-19 vaccination. The most stated reasons in the studies were conspiracy theories (Allagoa et al., 2021; Amuzie et al., 2021; Enitan et al., 2020; Olomofe et al., 2021; Tobin et al., 2021), followed by disbelief (Allagoa et al., 2021; Enitan et al., 2020; Uzochukwu et al., 2021), and queried vaccine safety (Adejumo et al., 2021; Adigwe, 2021; Allagoa et al., 2021; Iliyasu et al., 2021; Robinson et al., 2021). The other reasons are vaccine side effects (Adigwe, 2021; Iliyasu et al., 2021; Uzochukwu et al., 2021) and the fear of the unknown (Enitan et al., 2020; Olomofe et al., 2021; Robinson et al., 2021).

Discussion

COVID-19 continues to be a public health concern worldwide and to date~~, and it has not been controlled effectively~~. Different strategies have been put in place to combat the pandemic even as new strains of the virus keep emerging. One of the strategies is the development of the COVID-19 vaccine. Many effective vaccines have been developed and gone through clinical trials why so many are still under trial, yet to be approved. As of today, the COVID-19 vaccine is still the only effective means to control this pandemic and must be accepted by quite a number of the population to be able to combat the pandemic. Therefore, this review was intended to evaluate the acceptance rate of the COVID-19 vaccine among the general population in Nigeria.

~~This rapid review was done using a comprehensive search to include studies done in Nigeria including [illegible]VID-19 vaccination acceptance among the people. It was done using the PRISMA checklist. This is the first review done in Nigeria on the level of acceptance of the COVID-19 vaccine among Nigerians.~~ This information has significant implications for the government, researchers, healthcare policymakers and implementers, communities, and health care providers. This review showed that the estimate of the pooled prevalence of ~~acceptance rate of COVID-19 among Nigerians~~ ranges between 20.0%- 58.2% ~~vaccine acceptance rate~~. This finding ~~wa~~s consistent when compared with the studies conducted in other countries like Kuwait (53.1%), Palestine (40%), Malaysia (48.2%), England (55.8%), Greece (57.7%), and Saudi Arabia (48%). However, this finding was higher when compared with the two studies conducted in Jordan (36.8%), and Jordan (37.4%) (Wake, 2021). The difference could be due to the differences in the sociodemographic characteristics of the study population.

For the COVID-19 vaccine to be termed effective in Nigeria, it depends on a considerable level of acceptance ~~of the vaccine~~, which can improve the uptake among the general population ~~and then eventually develop~~ herd immunity. It has been suggested that about 70% of a population must have immunity either through a vaccine or previous infection for herd immunity to be achieved (MacDonald, 2015).

In this review, variability in the COVID-19 vaccine acceptance rate was found. However, certain patterns can be deduced based on the descriptive analysis of the reported vaccine acceptance rate or the period of ~~the conduct of the~~ study. Among healthcare workers, the acceptance rate was 55.5% in October 2020, 32.5% in January 2021, and 45.6% in March 2021. Among adults, the acceptance rate was 20.0% in May 2020, 58.2% in July 2020, 50.2% in August 2020, 51.1% in February 2021, and 45.6% in March 2021. This might be due to the differences in the sociodemographic characteristics of the study population, the time the study was conducted, and the awareness level of the study participants toward the COVID-19 vaccine at the time of the study.

The factors that were reported for non-acceptance of ~~the respondents to accept~~ COVID-19 vaccination were disbelief, lack of trust in the government, conspiracy theories, vaccine side effects COVID-19 fear of the unknown. These reasons are not surprising as they have been cited in various studies outside Nigeria as reasons for the non-acceptance of vaccines. (MacDonald, 2015; Marti, de Cola, MacDonald, Dumolard, & Duclos, 2017).

The result of this review demonstrated that the most reported factor in the studies were conspiracy theories that developed from misinformation, fake news, and political sagas radiating the internet in the process of the development of vaccines. In particular, the internet increased the audience for the anti-vaccine movement (Johnson et al., 2020), and has been able to influence a large population against COVID-19 vaccination. D~~isbelief~~ and lack of trust in the government concerning COVID-19 vaccines were also factors that were reported by the studies (Bish, Yardley, Nicoll, & Michie, 2011), ~~also reported that trust in authorities is associated with vaccination willingness~~. This might be as a result of the ~~fast production of the vaccines and the process of transaction and procurement of the vaccines from the developed countries~~ that brought about the phrase that “our government accepted them to use us Africans as guinea pigs to test their vaccines. About one million vaccines got expired due to a lack of uptake.

This review is the ~~first to evaluate the vaccine acceptance rate~~ and associated factors for the non-acceptance of the COVID-19 vaccine in Nigeria. However, it is a rapid review and is limited by the relatively few publications on both COVID-19 vaccine acceptance rate and predicting factors that may impact generalizability.

Conclusions and recommendations

Vaccines are the last hope, for now, to eradicate this deadly virus from the face of the earth. Nevertheless, the majority of the population are nay Sayers, vaccine doubters and so many conspiracy theories circulating that have led to non-acceptance of the vaccine. Hence, to improve vaccine acceptance in the country, there is ~~the~~ need for more context-specific research explicitly ~~aiming~~ to identify factors associated with vaccine hesitancy across the prevailing cultural, tribal, and religious tendencies (Ogundele, Ogundele, & Beloved, 2020).

The drivers for vaccine acceptance, no doubt will likely vary in different parts of the country, and therefore, it is evident from the Nigerian context that no single strategy can be used to improve vaccine acceptance in all the populations or regions of the country. Health-care providers must be informed enough on how to counter arguments against vaccines at all levels particularly because of vaccine distrust propagated by religious leaders in some sections of the country and be empowered with the message that the client will understand. There must be deliberate effort targeted at unvaccinated and under-vaccinated subpopulations and the hard-to-reach communities. There should be a deliberate emphasis on community-based approaches to increase the knowledge and awareness about vaccination (Ogundele et al., 2020), while directly engaging community leaders and gatekeepers and religious and/or other influential leaders to promote vaccination in the community. Also, to combat the widespread rumors and misinformation that has led to the non-acceptance of the COVID-19 vaccine, effective health messaging campaign must be put in place to encourage the acceptance of the vaccine. Policymakers, healthcare workers, and other stakeholders do more with regards to information dissemination and health education and promotion especially on the misconceptions about the COVID-19 vaccine.

Conflict of interest

The authors report no conflict of interest.

References

Adejumo, O. A., Ogundele, O. A., Madubuko, C. R., Oluwafemi, R. O., Okoye, O. C., Okonkwo, K. C., . . . Enikuomehin, A. C. (2021). Perceptions of the COVID-19 vaccine and willingness to receive vaccination among health workers in Nigeria. *Osong Public Health and Research Perspectives, 12*(4), 236.

Adigwe, O. P. (2021). COVID-19 vaccine hesitancy and willingness to pay: Emergent factors from a cross-sectional study in Nigeria. *Vaccine: X, 9*, 100112.

Allagoa, D. O., Oriji, P. C., Tekenah, E. S., Obagah, L., Njoku, C., Afolabi, A. S., & Atemie, G. (2021). Predictors of acceptance of Covid-19 vaccine among patients at a tertiary hospital in South-South Nigeria. *Int J Community Med Public Health, 8*(5), 2165-2172.

Amuzie, C. I., Odini, F., Kalu, K. U., Izuka, M., Nwamoh, U., Emma-Ukaegbu, U., & Onyike, G. (2021). COVID-19 vaccine hesitancy among healthcare workers and its socio-demographic determinants in Abia State, Southeastern Nigeria: a cross-sectional study. *The Pan African medical journal, 40*.

Bish, A., Yardley, L., Nicoll, A., & Michie, S. (2011). Factors associated with uptake of vaccination against pandemic influenza: a systematic review. *Vaccine, 29*(38), 6472-6484.

CDC. (2014). Public Health Guidance for Community-Level Preparedness and Response to Severe Acute Respiratory Syndrome (SARS).

Enitan, S., Oyekale, A., Akele, R., Olawuyi, K., Olabisi, E., Nwankiti, A., & Enitan, C. (2020). Assessment of Knowledge, Perception and Readiness of Nigerians to participate in the COVID-19 Vaccine Trial. *International Journal of Vaccines and Immunization, 4*(1), 1-13.

Iliyasu, Z., Umar, A. A., Abdullahi, H. M., Kwaku, A. A., Amole, T. G., Tsiga-Ahmed, F. I., . . . Aliyu, M. H. (2021). "They have produced a vaccine, but we doubt if COVID-19 exists": correlates of COVID-19 vaccine acceptability among adults in Kano, Nigeria. *Human vaccines & immunotherapeutics*, 1-8.

Johnson, N. F., Velásquez, N., Restrepo, N. J., Leahy, R., Gabriel, N., El Oud, S., . . . Lupu, Y. (2020). The online competition between pro-and anti-vaccination views. *Nature, 582*(7811), 230-233.

MacDonald, N. E. (2015). Vaccine hesitancy: Definition, scope and determinants. *Vaccine, 33*(34), 4161-4164.

Marti, M., de Cola, M., MacDonald, N. E., Dumolard, L., & Duclos, P. (2017). Assessments of global drivers of vaccine hesitancy in 2014—Looking beyond safety concerns. *PLoS One, 12*(3), e0172310.

NCDC. (2020). First case of Corona Virus Disease confirmed in Nigeria. Retrieved from https://ncdc.gov.ng/news/227/first-case-of-corona-virus-disease-confirmed-in-nigeria

NCDC. (2021). COVID-19 Nigeria. Retrieved from https://covid19.ncdc.gov.ng/

NPHCDA. (2021). COVID-19 vaccination update. Retrieved from https://nphcda.gov.ng/

Ogundele, O., Ogundele, T., & Beloved, O. (2020). Vaccine hesitancy in Nigeria: Contributing factors & way forward. *The Nigerian Journal of General Practice, 18*(1), 1-4. doi:10.4103/1008-682x.275508

Olomofe, C. O., Soyemi, V. K., Udomah, B. F., Owolabi, A. O., Ajumuka, E. E., Igbokwe, C. M., . . . Dada, O. F. (2021). Predictors of uptake of a potential Covid-19 vaccine among Nigerian adults. *medRxiv*, 2020.2012. 2028.20248965.

Robinson, E. D., Wilson, P., Eleki, B. J., & Wonodi, W. (2021). Knowledge, acceptance, and hesitancy of COVID-19 vaccine among health care workers in Nigeria. *MGM Journal of Medical Sciences, 8*(2), 102.

Sallam, M. (2021). COVID-19 vaccine hesitancy worldwide: a concise systematic review of vaccine acceptance rates. *Vaccines, 9*(2), 160.

Samaranayake, L., Chang, J. W. W., & Panduwawala, C. (2021). COVID-19 Vaccines: Vagaries and Vacillations. *Dental Update, 48*(4), 323-326.

Samaranayake, L., & Fakhruddin, K. S. (2021). COVID-19 vaccines and dentistry. *Dental Update, 48*(1), 76-81.

Tobin, E. A., Okonofua, M., & Azeke, A. (2021). Acceptance of a COVID-19 Vaccine in Nigeria: A Population-Based Cross-Sectional Study. *Annals of Medical and Health Sciences Research, 11*(5).

Uzochukwu, I. C., Eleje, G. U., Nwankwo, C. H., Chukwuma, G. O., Uzuke, C. A., Uzochukwu, C. E., . . . Esimone, C. O. (2021). COVID-19 vaccine hesitancy among staff and students in a Nigerian tertiary educational institution. *Therapeutic Advances in Infectious Disease, 8*, 20499361211054923.

Wake, A. D. (2021). The acceptance rate toward COVID-19 vaccine in Africa: a systematic review and meta-analysis. *Global Pediatric Health, 8*, 2333794X211048738.

WHO. (2015). *Middle East respiratory syndrome coronavirus (MERS-CoV): summary of current situation, literature update and risk assessment*. Retrieved from
WHO. (2021). Coronavirus (COVID-19) dashboard. Retrieved from https://covid19.who.int/

Click here to access/download
Supporting Information
Supplement- Prisma Checklist.docx
